# The Best Anaesthesia for Closed Reduction of Radial Fracture: Is the Jury Still Out?

**DOI:** 10.1111/aas.70084

**Published:** 2025-06-19

**Authors:** Johan Raeder, Axel R. Sauter

**Affiliations:** ^1^ Department of Anaesthesia and Intensive Care Medicine Oslo University Hospital Oslo Norway; ^2^ Faculty of Medicine, University of Oslo Norway; ^3^ Department of Anaesthesiology and Pain Medicine Bern University Hospital, Inselspital, University of Bern Bern Switzerland

**Keywords:** Colles' fracture, distal forearm fracture, haematoma block, median nerve block, radial nerve block, ultrasound guided nerve block

The paper of Christensen and co‐workers in the present issue of this journal [1] on choice of anaesthesia for reduction of Colles' radial fracture illustrates two fundamental questions in anaesthetic practice: (1) What is the simplest approach that is compatible with being able to do the surgical procedure? and (2) Does the choice of anaesthesia impact the success of the surgery?

A Colles' fracture is a very common injury [2], often diagnosed and treated immediately in the admission area with closed reduction, with the aim of ensuring satisfactory alignment of broken bones for consecutive conservative treatment with plaster cast. While general anaesthesia with muscle relaxation definitely will provide excellent conditions for the surgeons and unconsciousness for the patient, it is generally considered excessive in terms of resource use unless the case is complicated or there is an obvious need for open reduction.

Regional anaesthesia techniques, such as brachial plexus blocks, Bier blocks (intravenous regional anaesthesia) and distal peripheral nerve blocks, can be used as an alternative to general anaesthesia [3]. Although a plexus brachialis block can provide optimal pain relief and excellent surgical conditions, this method involves increased use of anaesthetic resources and carries some potential risks. On the other hand, simply providing sedation or intravenous conscious analgesia is usually too painful for the patient and may provoke muscle contraction, which may prevent the success of the closed surgical reduction. Thus, the use of a haematoma block is often used as a simple, first‐line anaesthetic procedure for this purpose. It can be applied immediately after the diagnosis is confirmed and can be performed by the present surgeon in charge, without involving anaesthetic personnel and subsequent extra measures of preparation and time consumption. As ultrasound techniques for identifying single peripheral nerves have developed during the past few decades, another possible solution is to just block the individual nerves (i.e., perform a nerve block), as was done in the study of Christensen and co‐workers [1]. A recent systematic review on the use of peripheral nerve blocks for closed reduction of distal radius fractures concluded that the evidence on the effect of peripheral nerve blocks was very limited [4]. Therefore, a well‐conducted clinical trial is highly warranted.

Then, where do we end up in terms of choosing between a haematoma block and a nerve block? While the data of Christensen and co‐workers do not provide a definite conclusion regarding the preferred choice, it does highlight some important aspects that should be taken into consideration. The primary endpoint, that is, satisfactory fracture reduction, was evaluated by blinded observers from the control x‐ray picture taken after fracture reduction and defined by fairly objective x‐ray criteria. The authors found a statistically significant difference in the satisfactory reduction rate in favour of nerve blocks (62%) versus haematoma blocks (40%). Surgical correction was performed on 52% of patients in the nerve block group and 66% of patients in the haematoma block group. However, the clinical significance of these results may be disputed. As the authors point out, the rate of satisfactory reduction, should be considered as a surrogate measure of clinical success, defined as no need of further surgical intervention during the subsequent 10 days. In both groups, one out of four patients were not subjected to further surgery despite of being classified as having non‐satisfactory reduction.

It may be surprising that the nerve block failed to provide better analgesia during the closed reduction manoeuvre. As much as 36% in this group reported pain at NRS > 7, and non‐significantly higher (*p* = 0.1) than the 25% in the haematoma block group. However, patients experienced significantly less pain during the application of nerve block: 15% of these patients reported an NRS score > 7, compared with 37% of the patients in the haematoma group. This may have been confounded by the use of concomitant analgesics in around half of the patients and by potential differences in non‐collected data on the time from admission until block performance. The haematoma block was performed by the surgeon in charge of receiving the patient, whereas the nerve block was performed by a junior anaesthesiologist, who probably had to be called upon for assistance. The delay from the application of the block until fracture reduction was, by default, a minimum of 15 min, but it took over an hour in one out of four patients in the nerve block group. The median time was 45 min in the nerve block group, as compared to 25 min in the haematoma group.

As fewer than 50% of the patients in the present study required additional surgery, both methods may have been favourable compared to the local Danish quality database material used for the statistical power calculation [1]. Only 15% of patients in the database did not need further surgery. However, this may be due to other issues, such as the diagnostic criteria used, the training of the surgeon, different logistics, and so forth; rather than the choice of anaesthesia.

Still, given the high ratings of pain in both groups, one may question whether any of the methods in the present study can be recommended. There is no data or discussion on whether the block method itself provides incomplete analgesia, or whether the somewhat disappointing group‐level results are due to some blocks being unsuccessful, while others were very appropriate. The authors raise the question of whether nerve block success would have been better with more experienced anaesthesiologists involved. The next obvious question is then: Why not do a better analgesic block, that is, an axillary or infraclavicular brachial plexus block? These blocks usually have a success rate of more than 90%, with very rare side effects when using a modern ultrasound approach [5, 6]. In the present study, the nerve block failure rate was at least 36%, as indicated by the number of patients with a pain score of seven or higher.

In conclusion, we are still unable to reach a definite conclusion on how this very common clinical fracture should be anaesthetised for primary reduction. However, the data from the large number of patients in the randomised prospective study of Christensen and co‐workers add important information on the debate and provides inspiration for further studies.

## Author Contributions

The authors, J.R. and A.R.S. have together developed the idea and the contents of the article and written the paper together.

## Ethics Statement

The authors have nothing to report.

## Conflicts of Interest

Axel Sauter serves as an Associate Editor for Acta Anaesthesiologica Scandinavica. No other competing interests.

## Data Availability

Data sharing is not applicable to this article as no new data were created or analysed in this study.

## References

[1] A. B. Christensen , C. IIkjær , K. LT , et al., “Ultrasound‐Guided Nerve Blocks Improve Success Rate of Closed Reduction of Colles' Fractures: A Randomised Controlled Trial,” Acta Anaesthesiologica Scandinavica (2025). 10.1111/aas.70063 PMC12178230 40536436

[2] U. G. Longo , S. De Salvatore , A. Mazzola , et al., “Colles' Fracture: An Epidemiological Nationwide Study in Italy From 2001 to 2016,” International Journal of Environmental Research and Public Health 20 (2023): 20. 10.3390/ijerph20053956 PMC10002201 36900966

[3] H. H. Handoll , R. Madhok , and C. Dodds , “Anaesthesia for Treating Distal Radial Fracture in Adults,” Cochrane Database of Systematic Reviews 2002 (2002): Cd003320. 12137688 10.1002/14651858.CD003320 PMC8713351

[4] S. Pisljagic , J. L. Temberg , M. T. Steensbæk , et al., “Peripheral Nerve Blocks for Closed Reduction of Distal Radius Fractures‐A Systematic Review With Meta‐Analysis and Trial Sequential Analysis,” Acta Anaesthesiologica Scandinavica 68 (2024): 1149–1160. 39039732 10.1111/aas.14474

[5] E. Albrecht , J. Mermoud , N. Fournier , C. Kern , and K. R. Kirkham , “A Systematic Review of Ultrasound‐Guided Methods for Brachial Plexus Blockade,” Anaesthesia 71 (2016): 213–227. 26670119 10.1111/anae.13347

[6] M. R. Jones , M. B. Novitch , S. Sen , et al., “Upper Extremity Regional Anesthesia Techniques: A Comprehensive Review for Clinical Anesthesiologists,” Best Practice & Research. Clinical Anaesthesiology 34 (2020): e13–e29. 32334792 10.1016/j.bpa.2019.07.005

